# Proposing a Robust Model to Reduce Employees’ Turnover Intentions in an Ethical Leadership Framework: Empirical Evidence from the Healthcare Sector

**DOI:** 10.3390/ijerph19158939

**Published:** 2022-07-22

**Authors:** Qiangzhen Jian, Xiuting Wang, Hisham Mohammad Al-Smadi, Aamer Waheed, Alina Badulescu, Sarminah Samad

**Affiliations:** 1 Wuhan Institute of Development Strategy, Wuhan 430070, China; yjfhjzq@163.com; 2 School of Management, Wuhan University of Technology, Wuhan 430070, China; 3 Department of Financial and Administrative Sciences, Ajloun College, AL-Balqa Applied University, Ajloun 26816, Jordan; dr-hsmadi@bau.edu.jo; 4 Department of Management Sciences, COMSATS University Islamabad (CUI), Islamabad 44000, Pakistan; awaheed@comsats.edu.pk; 5 Department of Economics and Business, Faculty of Economic Sciences, University of Oradea, 410087 Oradea, Romania; abadulescu@uoradea.ro; 6 Department of Business Administration, College of Business and Administration, Princess Nourah bint Abdulrahman University, Riyadh 11671, Saudi Arabia; sarminasamad@gmail.com

**Keywords:** leadership, mental health, turnover, healthcare system, organizational management

## Abstract

Employee turnover is expensive and disruptive for an organization. Studies have already mentioned that the economic cost of turnover is huge, ranging from 90% to 200% of the existing employee’s salary. With an increase in turnover rate, the social fabric of an enterprise may be disrupted. Additionally, organizations with an increasing turnover are expected to lose intangible knowledge and skills, operational effectiveness, customer satisfaction, and product or service quality. In a healthcare context, an increasing turnover rate has more consequences than other sectors because the healthcare sector worldwide is already identified as a sector facing resource scarcity. Exacerbating the situation, current evidence suggests that employee turnover has been increasing globally in the healthcare sector. The literature suggests that an ethical leadership style may reduce employees’ likelihood of quitting an organization. However, such literature is sparse in healthcare, especially from the perspective of a developing economy in the Global South, which is more resource-deficient than the Global North. To fill this knowledge gap, this study investigates the relationship between ethical leadership style and turnover intentions in the healthcare context of the Global South. This study also tests the mediating effect of intrinsic motivation and psychological contract fulfillment in the above-proposed relationship. Furthermore, the conditional indirect effect of resilience is also tested. The data are collected from the hospital employees through a self-administered questionnaire. The hypothesized relationships are tested through structural equation modeling. The empirical evidence indicates that ethical leadership reduces employees’ turnover intentions significantly. The results further confirm the mediating and moderating effects of intrinsic motivation, psychological contract fulfillment, and resilience. These results have different theoretical and practical implications for the healthcare sector. The results especially highlight the role of ethical leaders in a hospital to deal with the challenge of turnover, which has been rising worldwide.

## 1. Introduction

Researchers, academics, and organizational professionals have sought to understand the factors that force an employee to quit their job. Turnover is expensive and disruptive in any form (functional or dysfunctional). Studies have already mentioned that the economic cost of turnover is huge, ranging from 90% to 200% of the existing employee’s salary [1]. An increasing turnover rate has many negative consequences for an organization. For example, with an increase in turnover rate, the social fabric of an enterprise may be disrupted [2]. Additionally, organizations with an increasing turnover are expected to lose intangible knowledge and skills, operational effectiveness, customer satisfaction, and product or service quality [3]. Considering different negativities associated with turnover, contemporary enterprises from every sector of an economy place a great emphasis on retaining their employees and understanding the factors contributing to turnover. When linked to the healthcare system, an increasing turnover rate has more consequences than other sector because the healthcare sector worldwide is identified as a sector facing resource scarcity [4,5]. Studies have shown that turnover has been increasing in healthcare systems globally [6]. From an economic perspective, in a sector that already faces a shortage of resources, bearing the cost of employee turnover is something that makes an additional dent in healthcare resources. For example, a recent report shows that in the USA alone, a hospital bears an average loss of more than USD 4 million each year due to turnover. A similar trend was also reported in other countries (USD 48,790 in Australia and USD 26,652 in Canada) [7].

The above discussion is enough to infer that turnover is a critical challenge that the healthcare sector faces almost in every region of the world. Although turnover in healthcare has risen in the recent past, a recent study suggests that 75% of the reasons why employees quit their jobs could be prevented. The present document shows that one of the top three most common reasons for turnover is an inappropriate leadership behavior [8]. This indicates the importance of an effective leadership style in mitigating employees’ turnover intentions in a healthcare context.

Prior literature indicates that an ethical leadership style of management could reduce turnover intentions among employees. For example, it was noted that a corporate manager, as an ethical leader, could significantly reduce turnover in the banking [9,10] and hospitality sectors [11,12]. Although the role of ethical leadership in reducing the turnover intentions of employees was highlighted in prior literature at different levels and sectors, surprisingly, such investigations remained sparse in a healthcare context with some exceptions. Among such exceptions, we refer to the studies by Mayende and Musenze [13] and Hashemi et al. [14]. However, we feel this limited explanation is insufficient to advance this debate and to reach a consensus in a healthcare context. Therefore, more investigation is required in this area. This study aims to extend the debate on the relationship between ethical leadership and healthcare employees’ turnover intentions.

A growing body of knowledge has already indicated that turnover is a complex phenomenon that, in an organizational milieu, takes shape due to different personal, contextual, and organizational factors [3,15]. In this respect, the previous literature indicates the importance of different psychological factors as mediators in understanding the turnover intentions of employees in a certain leadership framework. For example, job satisfaction [16], commitment [17], citizenship behavior [18], and intrinsic motivation [19] were specified as psychological factors that mediate between a leadership style and turnover intentions. Among these factors, it was mentioned that an intrinsically motivated employee has less intention to quit a job. Literature also emphasizes that an effective leadership style can positively enhance employees’ intrinsic motivation, which can then buffer the negative effect of turnover [20]. Even some recent studies noted the mediating effect of intrinsic motivation in an ethical leadership framework [21]. Although employees’ intrinsic motivation can reduce turnover intentions, its key role was less highlighted in a healthcare context. Therefore, this study intends to bridge this gap by investigating the mediating effect of intrinsic motivation between ethical leadership and employees’ turnover intentions in a healthcare context.

At the same time, another important psychological mediator that influences employee behavior in an organizational milieu is the psychological contract fulfillment perceptions of employees in an organization. Specifically, the seminal work by Collins [22] indicated that when employees feel that the management of an organization fulfills their psychological contracts, they show positive behavior and trust such organizations, which ultimately reduces their turnover intentions. Especially in a healthcare system, psychological contract fulfillment has a special role because the healthcare field is not only physically demanding but also requires socioemotional aspects in employer–employee relationships [23]. The dynamic and uncertain working environment in healthcare makes it critical for healthcare managers to improve employee perceptions about psychological contract fulfillment [24] to reduce their intentions to leave a hospital. Therefore, it is worthwhile to investigate the mediation effect of psychological contract fulfillment between ethical leadership and turnover intentions.

Literature in the domain of positive psychology indicates that certain personality characteristics of an individual can significantly influence their behavior [25]. A growing body of knowledge has shown mounting interest in understanding resilience among different personality factors. The positive outcomes associated with resilience are not the only reasons recent organizational management scholars show a great interest in resilience to employee behavior influenced. The most important aspect of resilience is that it can be improved as it is influenced by different factors [26]. Studies suggest that in a healthcare context, resilient employees are better able to manage stressful situations and have better mental health [27,28], which ultimately improves their turnover intentions. Previous literature also emphasized that personality characteristics can act as potential moderators to predict certain behaviors. In this respect, the conditional indirect role of resilience in buffering negative outcomes of employees was recently discussed [29,30]. Extending this debate, this study intends to introduce resilience as a moderator between the relationship of ethical leadership and turnover intentions through intrinsic motivation and psychological contract fulfillment.

The target segment of this study is the healthcare sector of Pakistan, which is a developing country in the Global South. We selected this segment to test the proposed relationships in this study due to the following reasons. First, like in other regions of the world, turnover in the healthcare sector in Pakistan is a critical issue [31]. Hence, exploring the factors that can reduce turnover in this sector is worthwhile. Second, considering the economic cost associated with turnover, the healthcare sector in a Global South context presents a bleak picture. Unlike the Global North, the healthcare systems in most Global South countries face huge resource shortages [32]. In this respect, an increasing turnover rate further adds to the difficulty levels in this sector, which undermines the quality of healthcare service. Therefore, it is of utmost importance to reduce the turnover intention of employees in a Global South context.

Ultimately, this study advances the theoretical debate by bridging the following knowledge gaps. Firstly, this study is one of the scarce studies that highlight the role of ethical leadership in reducing healthcare employees’ turnover intentions. In this respect, most of the prior literature did not investigate this relationship by focusing on a healthcare system. Secondly, this study intends to advance the debate on turnover by adopting a more robust model, as the current study is the first—at least to our knowledge—that considers organizational factors (ethical leadership), psychological factors (intrinsic motivation and psychological contract fulfillment), and personality characteristics (resilience) to explain employees’ turnover intentions. Thirdly, the current study tends to contribute to the existing literature on turnover from a healthcare perspective of the Global South, whereas most of the previous studies were carried out in the Global North.

## 2. Theoretical Framework and Hypotheses

Self-concept theory has been recognized as one of the leading theories that help to anatomize the turnover intentions of employees, especially from an ethical perspective [33,34]. Defined by Rosenberg [35], self-concept is the totality of one’s feelings and thoughts regarding their perception of themselves. Shamir et al. [36] were among the pioneers who extended this concept in an organizational context. They argued that in an organizational setting, an employee’s perceptions about others significantly influence their own self-evaluation. From a leadership perspective, this theory indicates that an effective corporate leader is able to influence the behavior of employees by connecting with their self-concept and helping to link the self-concept of an individual with the organization’s mission and goals [37]. Indeed, this theory provides a theoretical justification for how the turnover intentions of employees are dependent, at least in parts, on a corporate leader [9]. Specifically, previous researchers have attempted to explore the link between ethical leadership and employee turnover with the help of self-concept theory [38,39]. We refer to the definition of Brown et al. [40] in defining ethical leadership, who stated that “ethical leadership is the demonstration of normatively appropriate conduct of a manager through personal actions and interpersonal relationship with the employees. At the same time, an ethical leader is the promoter of such ethical conduct among the employees via two-way communication, decision making and reinforcement.” In this definition, an ethical leader is one who improves the mental health of employees by providing them with a meaningful work experience and by linking their self-concept with the larger moral purpose of an organization. When there is congruence between the self-concept of employees and organizational goals, as an antecedent of ethical leadership, we expect that an employee will stay with stay with such an organization. Thus, this theory clearly helps to improve the theoretical understanding of employee–employer relationships from an ethical leadership perspective.

Leadership literature suggests that corporate leaders are in great positions to influence employees’ attitudes and behaviors [5,41,42]. While different leadership styles have been discussed previously to influence different employees’ outcomes, recently, the concept of ethical leadership has gained momentum. In this respect, different positive aspects of ethical leadership have been discussed by existing research [43,44]. Specifically, it was documented in the literature that when corporate leaders act ethically in an organization, employees positively evaluate such ethical conduct of their leader and show positive attitudes and behaviors, which ultimately reduces their turnover intentions [19,45]. Lin and Liu [9] mentioned that an ethical leader in an organization not only influences the positive attitudes and behaviors on the part of employees but also has a critical role in mitigating and diverting the undesired behavior of employees, including turnover intentions. On a further level, an ethical leader manifests the art of developing a shared ethical aspiration among their followers. At the same time, an ethical leader builds a strong interpersonal relationship with employees in an organization, reducing value conflicts and ultimately reducing employees’ turnover intentions [46]. Based on the principal assumption of self-concept theory, an ethical leader is expected to enhance the motivation level of the employees by creating a shared sense of ethics and by developing a positive ethical identity. Past researchers have extensively used this theory to explain employee outcomes in different leadership contexts [47,48,49]. Moreover, Lin and Liu [9] documented a negative link between ethical leadership and employees’ turnover intentions in the light of self-concept theory. They stated that to understand this mechanism, one must explore the relationship between ethical leadership and turnover intentions based on the theoretical underpinning of self-concept theory because the crux of this theory emphasizes that employees are expected to stay in an organization when they believe their leadership is ethical. Conversely, the bulk of the literature has suggested when employees find their leadership unethical, they have higher intentions to quit such organizations [50,51]. Therefore, the above literature can be summarized by stating:

**Hypothesis** **1** **(H1).** *The presence of an ethical leader in an organization reduces the likelihood of employees’ turnover intentions*.

Intrinsic motivation is defined as the process in which an employee is attracted to a task itself. Further, an intrinsically motivated employee is expected to engage in a certain task due to his or her inner motivation level rather than receiving direction from someone [52]. In this respect, previous research shows a positive relationship between ethical leadership and the intrinsic motivation of employees [53,54]. Indeed, an ethical leader provides assistance to their followers in understanding the meaning of their job by communicating with them the ethical values of an organization [55]. When employees understand the ethical values of their organization, a feeling of praise develops in themselves, which then raises their intrinsic motivation further [56,57]. Additionally, an ethical leader empowers his or her followers by seeking their input in corporate policy making. This not only promotes the confidence level of employees, but also causes them to feel themselves to be safe under the supervision of their ethical leader [58]. While the prior literature establishes a positive link between ethical leadership and intrinsic motivation, it was also mentioned that intrinsic motivation is a potential mediator in predicting the turnover intentions of employees [19,54]. Specifically, in a healthcare context, Wang et al. [59] indicated that healthcare employees show greater motivation for their job because they are intrinsically motivated to serve humanity due to feeling a moral obligation. In the presence of an ethical leader, employees’ intrinsic motivation is enhanced further, which then provides an added explanation for the negative effect of ethical leadership and turnover intentions. Moreover, referring to the self-concept theory, employees under an ethical leader develop this belief that their leadership and organization share the same values that they reflect in themselves. This feeling of value congruence enhances their intrinsic motivation, which then reduces the turnover intentions of employees. Prior literature also acknowledges the role of self-concept theory in influencing the intrinsic motivation of employees in an organizational setting [60,61]. Ultimately, the above discussion clearly leads to establish the following set of hypotheses.

**Hypothesis** **2** **(H2).** *Ethical leadership in an organization directly influences the intrinsic motivation of employees*.

**Hypothesis** **3** **(H3).** *Intrinsic motivation mediates between ethical leadership and turnover intention of employees*.

Argyris [62] and Solley et al. [63] were the first to introduce the concept of psychological contract fulfillment to describe the understanding between employees and their foreman as a result of a particular style of leadership. Rousseau [64] presented a definition of psychological contract fulfillment by stating that it is the beliefs of an individual regarding the terms and conditions of an agreement with an employer. Robinson [65] referred to psychological contract fulfillment as a set of unwritten promises and expectations between employee and employer. Since the emergence of the psychological contract concept, researchers have developed different models to understand different employee outcomes in the psychological contract framework. For example, to understand organizational citizenship behavior of employees [66], work behavior [67], role behavior [68], and various other employee–employer relationships. On one end, for employers, it is a process through which they can assess employees’ perceptions of their psychological contract type. This helps employees to assess whether their employer fulfills their agreement over a specific period of time. Ultimately, employees’ perceptions of psychological contract fulfillment can influence their attitudes, behaviors, and working intentions.

Previous research indicates that the perceptions of employees regarding psychological contract fulfillment may be determined by the level and extent to which their employer fulfilled perceived promises in the past. Furthermore, it can also be determined by the extent to which employees perceive that they were treated fairly by their leadership in the past and if they feel that this will be reflected by their employer in the future [69]. Recently, the research on psychological contracts has gained momentum as a way to understand employee behavior, especially with regards to turnover intentions [70,71]. Specifically, it was mentioned in the previous literature that employees’ perceptions of psychological contract breaches by their leader could give rise to their intentions to quit an organization [72,73]. In this respect, we argue here that in a healthcare context, the experience of healthcare employees with an ethical leader is likely to improve their perceptions of psychological contract fulfillment. This line of reasoning is evident in the work of Tseng and Wu [74] and Li et al. [75]. We further argue that an ethical leader is the promoter of trust in employee–employer relationships. Employees with an improved level of trust in their organization due to their ethical leader are expected to have a greater perception of psychological contract fulfillment, which reduces their intentions to leave an organization in the future [42]. Brown and Mitchell [76] suggested that in the presence of ethical leadership, employees feel less ambiguity about the fulfillment of the promises proposed by their employer. Moreover, with respect to self-concept theory, in an ethical organization, employees’ self-concept regarding the ethical conduct of their employer is improved, which also enhances their perceptions of psychological contract fulfilment [77]. Therefore, it is expected that in the presence of an ethical leader in an organization, employees’ perceptions of psychological contract fulfillment improve, which then reduces their turnover intentions. Hence, we propose the following hypotheses:

**Hypothesis** **4** **(H4).** *The existence of an ethical leader in an organization enhances the perceptions of employees regarding psychological contract fulfillment*.

**Hypothesis** **5** **(H5).** *Psychological fulfillment mediates between ethical leadership and turnover intentions*.

**Figure 1:**
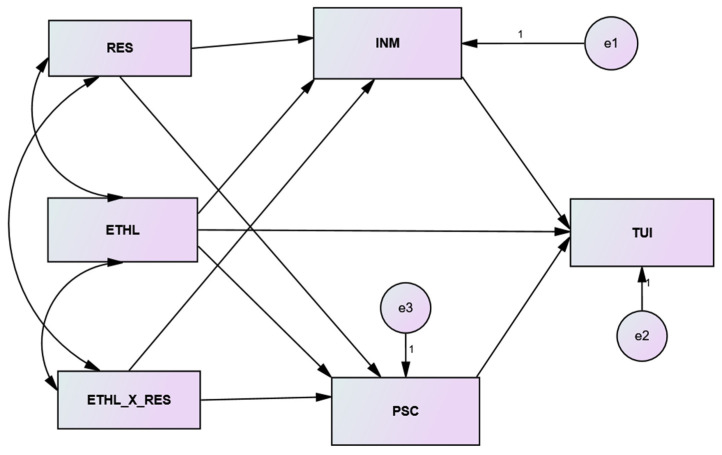
The hypothesized structural model: ETHL = ethical leadership (X); INM = intrinsic motivation (M1); PSC = psychological contract (M2); TUI = turnover intentions (Y); RES = resilience (W); ETHL_×_RES = interaction term.

Employee resilience has been discussed in prior literature on positive human psychology at many levels [78,79]. Zautra et al. [80] referred to resilience as a personality characteristic of an individual that helps them to deal with negative situations in an organization. Previous research mentions that resilience is a critical psychological factor that promotes mental health and employee well-being, which are of utmost importance to the success of an organization [81]. Luthar et al. [82] believed that resilience is an individual’s healthy and hopeful view of life in the face of different threats, traumas, and other social and physical problems. Individuals with high resilience values are expected to confront difficulties rather than quit or leave stressful situations. A notable aspect of resilience is that it is not necessarily an inborn characteristic of an individual. Rather, previous research indicates that resilience can be learned and improved over a lifetime [83]. Richardson [84], as well as Thies and Travers [85], believed that resilience varies between individuals and is dependent on different personal and organizational factors. In this respect, the available literature already indicates that an effective leadership style of a corporate leader could enhance employee resilience significantly [86,87]. In a healthcare context, the promotion of resilience among employees is of seminal importance because healthcare employees more often face different stressful situations which—if not managed—can lead employees to quit their jobs [88]. We argue here that an ethical leader enhances the level of resilience among employees through his or her ethical conduct in the workplace. An ethical leader sets the highest ethical standards in an organization based on morality, fairness, and care for all. Furthermore, as a promoter of ethics, an ethical leader enhances the level of trust of employees in leadership and organization. All these factors associated with ethical leadership influence employee resilience positively. Furthermore, as literature on positive psychology identifies resilience as a promoter of employee wellbeing and mental health [89,90]. It is expected that resilience as a moderator [91] in an ethical leadership framework will influence the intrinsic motivation and psychological contract fulfillment (factors related to positive human psychology) of employees, which then reduce turnover. Therefore, we expect that resilience will moderate the mediated relationship between ethical leadership and turnover. Please refer to Figure 1. Specifically, we hypothesize:

**Hypothesis** **6** **(H6).** *Resilience moderates between the mediated relationship of ethical leadership and turnover intentions through intrinsic motivation*.

**Hypothesis** **7** **(H7).** *Resilience moderates between the mediated relationship of ethical leadership and turnover intentions through psychological contract fulfillment*.

## 3. Methodology

### 3.1. Unit of Analysis, Sample, and Procedure

This study selected different hospitals from Lahore, which is the provincial capital of the largest province (Punjab) of Pakistan. The city constitutes a multi-million-person population, and a large number of hospitals operate in this city. Hospital staff in most hospitals face a overburdened situations often [92] because not only is the vast population of Lahore dependent on these hospitals for healthcare facilities, but patients from other remote areas also visit hospitals in Lahore. In terms of administrative structure, hospitals are operated by both the government and the private sector. It has been reported that the country faces a difficult situation in terms of healthcare delivery facilities. Poor infrastructure, alarming physician-to-patient and nurse-to-patient ratios, inappropriate management, and several other factors contribute to the poor health facility conditions in the country. Indeed, Pakistan is placed 154th among 190 countries in terms of healthcare facilities, which shows how resource-deficient this sector is [93]. To worsen the situation, if the economic cost of turnover is considered, the situation becomes bleaker. Therefore, this study tends to help the healthcare sector of Pakistan deal with the turnover challenge through an effective (ethical) leadership style.

We contacted different hospitals to ask for their support and favor in the process of data collection. A total of five hospitals responded positively to our request for data collection. Thus, we visited these hospitals to make direct contact with the employees. We visited these hospitals on different days, times, and shifts (day/night) so that a diverse umbrella of employees could participate in this survey. Therefore, the unit of analysis in this study was the individual employees who act as the respondents in this study. Specifically, employees from different departments and ranks were invited to participate in this survey. Data collection was completed in six weeks (December 2021 to January 2022).

### 3.2. Instrument

A questionnaire was prepared to collect data from different respondents in this survey. The items to measure the main variables in this study were adapted from different published sources (detail is provided in a later section). The preliminary version of the questionnaire was presented to experts in the field and academia for their expert assessment [94,95,96]. Once the questionnaire was evaluated by the experts, the final version was prepared, which was then given to each respondent in this study. Generally, the questionnaire included two major sections. Socio-demographic information was collected in the first section, whereas the variable-related responses were collected in the second section on a five-point Likert scale. Furthermore, following the major ethical protocols given in the Helsinki Declaration [97,98,99,100], we provided an informed consent form to each respondent, which indicated that this was volunteer activity and did not involve any personal risk. On a further note, the respondents’ information was kept anonymous.

The data were collected in two waves to reduce the fatigue and social desirability on the part of respondents. Each administered with a gap of two weeks between them. Mainly, socio-demographic information and employees’ perceptions regarding ethical leadership (ETHL) were collected in the first wave. Information related to turnover intentions (TUI), intrinsic motivation (INM), psychological contract fulfillment (PSC), and resilience (RES) was collected in the second wave.

### 3.3. Sample Size and Data Cleaning

To calculate the sample size of this study, we used a priori sample size calculator developed by Dniel [101]. This calculator is specially designed to be applied to structural equation modeling (SEM). Furthermore, the ability of this calculator to determine the minimum sample size for a specific study makes it an attractive tool in the eye of contemporary social sciences data analysts [102,103]. Specifically, this calculator estimates a sample size based on the input information, including the number of observed and unobserved variables in a study, the expected effect size, and the probability level. Applying this to the current study (observed variables = 32, unobserved variables = 6, probability level 95%, and with a medium effect size), the calculator indicates that the minimum sample size for this study should be 246. Considering the low response rate issue in most consumer- and employee-based surveys, we distributed 500 questionnaires among the selected hospital employees to achieve a sample size beyond 246. We received back 366 questionnaires, among which some were not included in the final dataset, as they were either partially filled or had some other issues (outliers). The final dataset included 342 surveys that were fully filled and did not include any cases of outliers. For further detail, Table 1 can be seen in which we see some detail on data cleaning. Furthermore, we employed a Mahalanobis technique to detect the cases of outliers using AMOS software (Table 2). The output indicated that 11 cases should be removed, as they significantly deviated from the mean (*p* < 0.05).

Both male and female respondents were included in this survey. However, we received a some higher response rate from female respondents (54%). The ages of the respondents were between 18 and 60 years. Most of the respondents were between the ages of 18 and 45 years (92%). Lastly, the experience level varied from 1 to above 10 years. However, the majority of the respondents fell between 1 and 7 years of experience.

### 3.4. Measures

To measure five variables in this study (ETHL, TUI, INM, PSC, and RES), we adapted items from different reliable published sources. The predictor variable (ETHL) was measured by adapting 10 items developed by Brown et al. [40] (items include: “My manager/leader discusses business ethics or values with employees”, “My manager/leader sets an example of how to do things the right way in terms of ethics”, “My manager/leader listens to what employees have to say”, and “My manager/leader conducts his/her personal life in an ethical manner”). Similarly, the criterion variable (TUI) was measured by using 4 items developed by Kelloway et al. [104] (items include: “I am thinking about leaving this organization”, “I am planning to look for a new job”, and “I intend to ask people about new job opportunities”).

There were two mediating variables in this study (INM and PSC), which were measured by using the scales of Tierney et al. [105] and Robinson and Wolfe Morrison [106]. Specifically, the scale of INM consisted of 5 items (items include: “I enjoy creating new procedures for work tasks” and “I enjoy finding solutions to complex problems”), and the scale of PSC included 7 items (items include: “So far my employer has done an excellent job of fulfilling its promises to me”, “I feel that my employer has come through in fulfilling the promises made to me when I was hired”, and “I have received everything promised to me in exchange for my contributions”). Lastly, the moderating variable (RES) was measured by using a 6-item scale from Smith et al. [107] (items include: “I tend to bounce back quickly after hard times” and “It does not take me long to recover from a stressful event”). The interitem consistency (Cronbach alpha-α) for each variable was significant ((ETHL = 0.912, TUI = 0.796, INM = 0.840, PSC = 0.897, and RES = 0.880).

### 3.5. Common Method Bias

Because the data were collected from a single source in this survey (employees), there may exist the issue of common method bias (CMB). To verify if this issue affects the quality of our data, we performed a common latent factor (CLF) test in AMOS. In doing so, we developed two measurement models. The first model was the actual conceptualized model, whereas in the second model, we introduced a CLF. The results of both models were assessed to detect any significant variance (greater than 0.2) in the standardized regression weights. It was noted that both models produce similar results (with slight differences). This indicates CMB was not a critical issue in this study. Moreover, the items of variables were randomly scattered on the questionnaire to break a sequence (if any) of respondents in answering the questions. Moreover, the respondents were also informed that their true response was very important for this research study. Additionally, we used simple questions so that there is no ambiguity on the part of respondents in answering a question. All these steps were helpful in reducing the issue of CMV [108,109,110,111].

## 4. Results

### 4.1. Reliability and Validity

We started the data analysis phase of this study by verifying the validity and reliability of each variable (ETHL, TUI, INM, PSC, and RES). In this vein, we first checked the convergent validity for which the standardized factor loadings of each variable’s item were assessed. For this purpose, the hypothesized measurement model was developed in the first stage. The purpose of the model development activity was to check if an item with weak factor loadings exists. The output of the measurement model is reported in Table 3, where it can be seen that the item loadings of most variables were significant (≥0.7), except for one ETHL item which showed poor factor loading (0.44). We deleted this item from further analysis and continued our data analysis with nine ETHL items.

Based on these item loading values, we were able to calculate the value of average variance extracted (AVE) for all cases. It was noted that these values were greater than 0.5 in all cases, which indicates that the convergent validity was significant in each case. Specifically, AVEs ranged from 0.526 (INM) to 0.571 (ETHL). After verifying the convergent validity of all variables, we tested composite reliability in the second stage of the data analysis. The same previous factor loadings served as a base to calculate the composite reliability values. It was observed that the convergent validity in all cases was significant (>0.7). Specifically, the values ranged from 0.820 (TUI) to 0.923 (ETHL).

### 4.2. Model Fitness

In the next phase of data analysis, we developed different measurement models with different compositions (details are given in Table 4). The purpose of this exercise was to statistically assess which theoretical model fits best to the data. In this vein, four measurement models were developed, of which model 1 was the actual hypothesized (five-factor) model, and model 4 was a one-factor model. Models 2 and 3 were developed by combining different variables to produce a three-factor model (Model 3) and a two-factor model (Model 2). To arrive at a decision, we observed the different model fit indices of all four models. Specifically, normed fit index (NFI) and comparative fit index (CFI) were assessed against their standard acceptable values. Furthermore, we also evaluated these models based upon chi-square/degree of freedom and root mean square errors of approximation (RMSEA) values. This activity revealed that a one-factor model fits the data poorly, whereas a five-factor model (model 1) was superior in all cases (NFI = 0.963, CFI = 0.962, *χ*^2^*/df* = 1.989, and RMSEA = 0.047).

### 4.3. Correlations

Table 5 includes the output of correlation analysis, discriminant validity, mean values of variables, and standard deviation values (SD). In this respect, the highest mean value was observed in the case of RES, which showed a mean value of 3.146. Similarly, the SD values ranged from 0.548 to 0.638. Regarding the correlation values (*r*), we observed mixed results. For example, the *r* value between ETHL and TUI was negative (−0.478), indicating that ETHL and TUI are negatively related to each other. Similarly, a positive case can be observed in different cases—for example, between ETHL and RES (0.286) and between INM and PSC (0.362). Nevertheless, no value showed an extreme case (between −0.8 to 0.8). This implies that a multicollinearity issue was not critical in this research study. Lastly, to assess the discriminant validity, we compared the square root values of AVE for a variable with the correlation values. The results showed that the square root values of AVE were superior in all cases, indicating that the items of one variable were not alike compared to the items of another variable in any case.

### 4.4. Hypotheses Evaluation

The hypothesized relationships were tested by using structural equation modeling (SEM). The AMOS software was used for a complex second-generation multivariate data analysis solution for a covariance-based SEM . Along with AMOS, we also sought the guidance from the PROCESS plugin in SPSS [112]. This plugin enables data analysts to calculate different paths through differential equations. For this purpose, a model 7 formula was followed to build a user-defined syntax in the AMOS interface. Prior to this stage, we converted ETHL and RES into mean-centered variables, which is especially recommended for a model which includes moderating variables. After obtaining the mean-centered variables (ETHL and RES), we developed an interaction term (ETHL_X_RES) to produce an effect on two mediators (INM and PSC). Bootstrapping was also enabled by using a larger bootstrapping sample of 5000 [111]. The output of this analysis is given in Table 6, which shows different relationship values extracted from AMOS. It was noted that ETHL negatively predicted TUI, which is in line with the theoretical statement of H1. Specifically, it was noted that ETHL reduces the TUI of employees, indicating that H1 of this study should be accepted based on statistical evidence. The same type of argument may be repeated to infer that the statistical data also support the theoretical statements of H2 and H4.

The bootstrapping results establish that H3 and H5 were accepted, as they significantly mediated between ETHL and TUI. Specifically, it was noted that the inclusion of INM and PSC as mediators between ETHL and TUI significantly reduces the TUI of the employees. The nonzero point between any values of confidence interval (lower and upper) indicates that the mediation effects were significant in each case [112,113,114].

Lastly, to test the conditional indirect effect of RES between ETHL and INM and between ETHL and PSC, we tested the effect of RES at three levels of the mean. In this respect, the moderating effect of RES was tested 1 SD above the mean, at the mean, and 1 SD below the mean. The highest values achieved in this regard for moderating effect are reported in Table 6. The results indicate that the existence of RES as a moderator produces a further buffering effect between the mediated relationship of ETHL and TUI through INM and PSC. This clearly indicates that RES produces a significant conditional indirect effect. Thus, H6 and H7 were also accepted.

## 5. Discussion

This study confirms that the existence of an ethical leader in a hospital reduces the turnover intentions of employees (beta value = −0.4136) significantly. The ethical orientation of a corporate leader gives added support to the employees, due to which they feel elevation of trust, faith, and positive hope. All these factors eventually help the employees to stay with an organization for a long period of time, which ultimately reduces their likelihood of quitting that organization in the near future. Furthermore, an ethical leader not only influences the positive attitudes and behaviors of their followers, but they may significantly avert the undesired behavior of employees, including turnover intentions. Similarly, an ethical leader is capable of developing and promoting a shared ethical aspiration among their followers. Strong interpersonal relationships built by an ethical leader with employees also help them reduce value conflicts, which ultimately buffers their negative thoughts and intentions—for example, thinking about leaving an organization. Lastly, referring to the self-concept theory, an ethical leader is expected to enhance the motivation level of the employees by creating a shared sense of ethics and by developing a positive ethical identity, motivating them to stay in an organization as long as possible. This study confirms the findings in the previous literature that ethical leadership negatively predicts the turnover intentions of employees in an organization [9,14,45].

Another important finding of this study is confirmation of the mediating role of intrinsic motivation and psychological contract fulfillment in the relationship between ethical leadership and turnover intentions of employees. In this respect, the empirical evidence confirms that the intrinsic motivation level of employees in an ethical leadership framework provides them with the extra strength to cope with negative feelings in a workplace context. Past literature establishes a positive association between ethical leadership and the intrinsic motivation of employees [53,54]. An ethical leader helps employees to understand the meaning of their job task by communicating with them the ethical values of an organization. When employees understand the ethical orientation of their organization, they are infused with feelings of praise which then raise their intrinsic motivation. Furthermore, employees with better intrinsic motivation levels are less likely to quit an organization. Thus, the mediating effect of intrinsic motivation was significant in this study (beta value = −0.1242). These findings are in line with previous literature [19,115].

In the same vein, the mediating effect of psychological contract fulfillment was also supported by the empirical data. This finding is in agreement with previous research [69]. Employees’ perceptions of psychological contract fulfillment may be determined by the level and extent to which their employer fulfilled perceived promises in the past. Moreover, it can also be determined by the extent to which employees think that they were treated fairly by their leader in the past and if they feel the same will be reflected in the future. Therefore, in a healthcare context, the experience of healthcare employees with an ethical leader is likely to improve their perceptions of psychological contract fulfillment [74]. Furthermore, as the promoter of trust in employee–employer relationships, an ethical leader enhances the level of trust among their followers. In an organizational milieu, employees with an improved level of trust in their organization due to their ethical leader are expected to have a greater perception of psychological contract fulfillment, which reduces their intentions to leave an organization.

Lastly, this study also confirms that resilience acts as a moderator between the mediated relationship of ethical leadership and turnover intentions through intrinsic motivation (beta value = −0.1060) and psychological contract fulfillment (beta value = −0.1214). Past research has already mentioned that an effective leadership style of a corporate leader could enhance employee resilience significantly [86,87]. In a healthcare context, the promotion of resilience among employees is of seminal importance because healthcare employees more often face different stressful situations which, if not managed, can lead employees to quit their jobs. An ethical leader enhances the level of resilience of employees through his or her ethical conduct in the workplace. An ethical leader sets the highest ethical standards in an organization based on morality, fairness, and care for all. Thus, an ethical leader influences employees’ resilience positively, which then buffers the mediated relationship between ethical leadership and turnover intentions of employees.

### 5.1. Theoretical Contribution

This study contributes to the existing body of knowledge in the following ways. First, this study is one of the limited studies that discuss the role of ethical leadership in reducing healthcare employees’ turnover intentions. Though the relationship between ethical leadership and turnover intentions exists in prior literature [9,116], most of such literature did not investigate this relationship by focusing on a healthcare system. Specifically, this study extends the theoretical model developed by Lin and Liu [9] who highlighted the role of ethical leadership in reducing turnover intentions. However, they missed noticing the role of a personality characteristic (resilience, for example) in this relationship. This research offers a contribution similar to the conceptual model by Shareef and Atan [19], in which the authors highlighted the mediating role of intrinsic motivation but did not notice the moderating effect of resilience. Second, this study tends to advance the debate on turnover by adopting a more robust model, as the current study is the first—at least to our knowledge—that considers organizational factors (ethical leadership), psychological factors (intrinsic motivation and psychological contract fulfillment), and personality characteristics (resilience) to explain employees’ turnover intentions. Third, the current study tends to contribute to the existing literature on turnover from the perspective of healthcare in the Global South, whereas most of the previous studies were carried out in the Global North.

### 5.2. Practical Contribution

This study offers some practical insights into the healthcare sector, especially in the context of Pakistan. Considering the economic cost of turnover, which ranges from 90% to 200% of the cost of an existing employee, it is worthwhile to investigate factors that can reduce the turnover intentions of employees in an organization. The economic aspect of turnover has a special meaning to the healthcare system because it already faces resource insufficiency at a global level. In this vein, this research helps the healthcare sector by proposing an effective management style—for example, ethical leadership—to mitigate the negative outcomes of turnover in this sector. Specifically, by promoting an ethical leadership style, a hospital is expected to significantly reduce the turnover likelihood of employees. Additionally, from the perspective of Pakistan (a Global South country), where the healthcare system faces more resource scarcity compared to Global North countries, reducing employee turnover is an issue of seminal importance in which ethical leadership has a clear role.

Similarly, another key takeaway of this study is the recognition of personal factors, such as resilience and intrinsic motivation, in diverting undesired attitudes and behavior of employees. In this respect, a hospital can boost the level of intrinsic motivation and resilience of its employees by opting for an ethical style of organizational management. Especially referring to the earlier text in the onset of this document in which it was mentioned that 75% of turnover intention reasons could be prevented, it is very important for a hospital to pay attention to the organizational and personal factors that can help an employee to improve their positive feelings of wishing stay with a hospital as long as possible. When linked to the healthcare context of Pakistan, such findings have special importance because, like other regions in the world, the healthcare sector in the country also faces high employee turnover. In this respect, a specific hospital may reduce employee turnover by fostering resilience and intrinsic motivation in a leadership framework.

### 5.3. Limitations and Possible Future Directions

This study faces some potential issues which may be recognized as limitations. The first limitation of this study rests with the geographical consideration, as the current study collected the data only from Lahore. Though considering a large number of hospitals and considering the overburdened situation in most hospitals in Lahore, it was worth collecting the data from hospital employees in this city, we still suggest including more cities in order to achieve a better generalizability claim for this research. Another potential issue rests with the nature of the data. The current survey was conducted by following a cross-sectional survey design in which information was only collected at a specific point in time. Although cross-sectional surveys are very common in behavioral studies, establishing causal relationships under a cross-sectional survey method is difficult. Therefore, we suggest employing a longitudinal data design in future studies. Additionally, a non-probability sampling method was another potential limitation of this survey. Given that due to different policy and safety issues, most hospitals did not agree to share with us any list of employees, which could serve as a sampling frame to apply a probability sampling, we were unable to introduce any probability sampling technique. There is not any doubt in believing that probability sampling is regarded as superior compared to non-probability sampling. Therefore, if possible, we suggest that future studies subscribe to any probability sampling (for example, random sampling) method. On a further note, it is suggested that in future studies, different environmental factors, employees’ motivation and the issue of employability should be included in the proposed model of this study. Moreover, similar research should be carried out in other sectors or countries to guarantee the repeatability and thus the correctness of this logical thinking.

## 6. Conclusions

Ultimately, this research provides up-to-date insight into the healthcare sector of Pakistan to aid in combating turnover intentions of employees, which is reported as a rising phenomenon in a healthcare context, even at a global level. Because current evidence suggests that one of the top three reasons why employees leave their job lies with an inappropriate managerial style, it is very important for healthcare management to promote an ethical style of leadership. In this respect, we recommend that healthcare policymakers design special training and development programs for their managers and supervisors to indicate to them the importance of ethical leadership for effective organizational management. We also suggest such training programs at the employee level—we suggest arranging different seminars and workshops to improve the intrinsic motivation and resilience levels of employees. Furthermore, we also conclude that a hospital should provide clear communication to the employees through leadership that all the promises will be fulfilled by the organization honestly because when employees have a firm belief that their psychological contract will not be breached by the organization, it improves their mental health, which is important to reducing their turnover intentions. To conclude, we suggest considering ethical leadership as a style of effective hospital management to deal with the challenge of employee turnover, which has increased in the healthcare sector all around the world.

## Figures and Tables

**Table 1:** Data cleaning, outliers, and response rate.

	Distributed	Returned	Unreturned	Unusable	Outliers	Final
	500	366	134	24	13	342
Percentage	-	73.20	26.80	6.55	3.55	68.40

**Table 2:** Detection of Outliers.

Observation Number	Mahalanobis D-Squared	p1	p2
60	22.757	0.000	0.001
50	21.753	0.000	0.000
264	21.026	0.000	0.000
283	15.461	0.004	0.002
39	13.662	0.008	0.020
243	13.472	0.009	0.010
118	13.019	0.011	0.004
71	12.570	0.014	0.005
29	11.424	0.022	0.028
276	10.506	0.033	0.106
156	10.445	0.034	0.076
288	10.260	0.036	0.026
386	10.189	0.037	0.018

**Table 3:** Validity and reliability.

	λ	λ^2^	E-Variance
ETHL			
	0.722	0.521	0.479
AVE = 0.571	0.810	0.656	0.344
CR = 0.923	0.699	0.489	0.511
	0.705	0.497	0.503
	0.702	0.493	0.507
	0.739	0.546	0.454
	0.765	0.585	0.415
	0.819	0.671	0.329
	0.827	0.684	0.316
TUI			
AVE = 0.532	0.711	0.506	0.494
CR = 0.820	0.726	0.527	0.473
	0.702	0.493	0.507
	0.777	0.604	0.396
INM			
	0.738	0.545	0.455
AVE = 0.526	0.719	0.517	0.483
CR = 0.847	0.722	0.521	0.479
	0.706	0.498	0.502
	0.742	0.551	0.449
PSC			
	0.762	0.581	0.419
AVE = 0.552	0.719	0.517	0.483
CR = 0.896	0.718	0.516	0.484
	0.820	0.672	0.328
	0.703	0.494	0.506
	0.720	0.518	0.482
	0.752	0.566	0.434
RES			
	0.869	0.755	0.245
AVE = 0.610	0.714	0.510	0.490
CR = 0.903	0.716	0.513	0.487
	0.828	0.686	0.314
	0.702	0.493	0.507
	0.838	0.702	0.298

Notes: λ = Item loadings, CR = composite reliability, ∑λ^2^ = sum of square of item loadings, E-Variance = error variance.

**Table 4:** Model fit comparison, alternate vs. hypothesized models.

Model	Composition	*χ^2^*/*df* (<3)	Δ*χ*^2^/*df* -	NFI (>0.9)	CFI (>0.9)	RMSEA (<0.08)
1	(hypothesized) ETHL, TUI, INM, PSC, RES	1.989	-	0.963	0.962	0.047
2	(3-factor) ETHL + TUI + INM, PSC, RES	5.038	3.049	0.728	0.720	0.077
3	(2-factor) ETHL + TUI + RES, INM + PSC	5.863	0.825	0.613	0.613	0.109
4	(1-factor) TL + RLC + RSL + IMO + BO	7.653	1.790	0.462	0.448	0.163

**Table 5:** Correlations and discriminant validity.

Construct	ETHL	TUI	INM	PSC	RES	Mean	SD
ETHL	0.756	−0.478	0.502	0.392	0.286	2.793	0.638
TUI		0.730	−0.422	−0.526	−0.416	3.058	0.589
INM			0.722	0.362	0.469	2.635	0.646
PSC				0.743	0.228	2.969	0.610
RES					0.781	3.146	0.548

Notes: S.D = standard deviation, diagonal = discriminant validity values, *p <* 0.001.

**Table 6:** Hypotheses testing.

Hypotheses	Estimates (SE)	*t*/*z*	*p*-Value	CI
(ETHL→TUI)	−0.4136 (0.0462)	−8.9523	0.004	−0.521, −0.398
(ETHL→INM)	0.2683 (0.0492)	5.4532	0.000	0.293, 0.366
(ETHL→PSC)	0.2862 (0.0382)	7.4921	0.000	0.182, 0.428
(INM→TUI)	−0.4630 (0.0582)	−7.9553	0.007	−0.592, −0.388
(PSC→TUI)	−0.5582 (0.0521)	−10.7140	0.000	−0.679, −0.421
Mediating effects (ETHL→INM→TUI)	−0.1242 (0.0288)	−4.3132	0.000	−0.196, −0.102
(ETHL→PSC→TUI)	−0.1597 (0.0310)	−5.1534	0.000	−0.210, −0.143
Conditional indirect effect when INM is mediator	−0.1060 (0.0069)	−15.3623	0.000	−0.148, −0.111
Conditional indirect effect when PSC is mediator	−0.1214 (0.0097)	−12.5154	0.000	−0.142, −0.104

Notes: CI = 95% confidence interval with lower and upper limits.

## Data Availability

The data of this work will be provided on a reasonable request.
